# Clinical trial budgeting approaches in Switzerland—a meta-research study

**DOI:** 10.1186/s13063-025-08855-1

**Published:** 2025-05-14

**Authors:** Alexandra Griessbach, Malena Chiaborelli, Klaus Ehrlich, Regina Grossmann, María De Medina Redondo, Aurélie Fayet, Reinhard Maier, Sven Trelle, Angèle Gayet-Ageron, Alessandro Ceschi, Benjamin Speich, Matthias Briel, MAking Randomized Trials Affordable (MARTA) Group

**Affiliations:** 1 CLEAR-Methods Center, Division of Clinical Epidemiology, Department of Clinical Research, University Hospital Basel, University of Basel, Basel, Switzerland; 2 Swiss Tropical and Public Health Institute, University of Basel, Basel, Switzerland; 3 Department of Clinical Research, University Hospital Basel and University of Basel, Basel, Switzerland; 4 Clinical Trials Center, University Hospital Zurich, Zurich, Switzerland; 5 University Hospital Lausanne and University of Lausanne, Lausanne, Switzerland; 6 Clinical Trials Unit, Kantonsspital St. Gallen, Medizinisches Forschungszentrum, St. Gallen, Switzerland; 7 Department of Clinical Research, Clinical Trials Unit Bern, University of Bern, Bern, Switzerland; 8 Centre de Recherche Clinique, Methodological Support Unit, Geneva University Hospitals & University of Geneva, Geneva, Switzerland; 9 Clinical Trial Unit, Medical Education and Research Area, Ente Ospedaliero Cantonale, Lugano, Switzerland; 10 Division of Clinical Pharmacology and Toxicology, Institute of Pharmacological Sciences of Southern Switzerland, Ente Ospedaliero Cantonale, Lugano, Switzerland

**Keywords:** Clinical trials, Clinical trial budgeting, Trial funding, Trial costs

## Abstract

**Background:**

Conducting clinical trials is resource demanding. Mirroring challenges experienced elsewhere, clinical trials conducted in Switzerland often face overoptimistic budget estimations and insufficient funding, leading to trial discontinuation and research waste. As a first step to address this problem, we investigated the current approaches to estimate clinical trial budgets in Switzerland.

**Methods:**

We collected and examined the budgeting tools and approaches for clinical trials provided by the seven Swiss clinical trial units (CTUs) and the Swiss National Science Foundation (SNSF). We compared available approaches to the publicly accessible budgeting tool of the Belgian Health Care Knowledge Centre (KCE). For each approach, we collected data about user-testing, the availability of prespecified cost items, and estimates on cost ranges.

**Results:**

We found substantial heterogeneity in budget calculation approaches used by Swiss CTUs. None of the currently used tools and approaches provided by the seven CTUs or the SNSF was user-tested and neither supplied cost ranges for investigators to rely on. Five CTU tools included a detailed list of cost items. The SNSF provided a costing template with broad categories and is available for open grant applications only. One CTU tool was publicly available. The publicly available Belgian KCE tool was developed with user feedback and provided a detailed list of cost items, some cost ranges, and an instruction manual.

**Conclusion:**

Stakeholders should consider improving budgeting practices in Switzerland by standardizing cost items and user-testing approaches. The continuously improved Belgian KCE tool could provide orientation.

**Supplementary Information:**

The online version contains supplementary material available at 10.1186/s13063-025-08855-1.

## Introduction

Conducting clinical trials poses numerous challenges that are time and resource demanding, therefore leading to high costs [1, 2]. Overoptimistic, i.e., too low budget estimations, inadequate planning, and tight financial resources are common and increase the risk for premature trial discontinuation [3, 4]. Hereby, scarce research resources may be wasted. Clinical trials conducted in Switzerland are no exception [5, 6]. In interviews with trial investigators, funders, clinical trial support organizations, and ethics committees, we found that Swiss investigator-initiated trials are frequently underfunded or poorly budgeted [7]. Consequently, tools to support investigators in the careful planning and budgeting of clinical trial costs are instrumental for the successful conduct of trials and to avoid research waste.

A recent scoping review showed that freely available, user-tested, and validated budget planning tools for clinical trials are lacking [8]. In Switzerland, clinical trials units (CTUs) support clinical researchers with the planning of their clinical trials in addition to assistance with grant applications. The Swiss National Science Foundation (SNSF) is the main public funder for investigator-initiated trials in Switzerland. It is responsible for evaluating submitted trial proposals including budget estimates. In the present study, we aimed to examine and characterize the tools and approaches employed by Swiss CTUs and the SNSF when calculating clinical trial budgets and to compare them to a freely available international tool [9, 10] that was identified in a scoping review [8].

## Methods

This study is reported following the Standards for Reporting Qualitative Research (SRQR) guideline [11].

### Data collection

This meta-research study was conducted between March 2023 and December 2023. We contacted the seven Swiss CTUs within the Swiss Clinical Trial Organisation CTU Network (Department Klinische Forschung Basel, CTU Bern, Centre de Recherche Clinique Geneva, Clinical Research Center Lausanne, La Clinical Trial Unit dell’Ente Ospedaliero Lugano, CTU St. Gallen, and Clinical Trial Center Zurich) and asked them to share their current budgeting approaches. Additionally, we considered the SNSF clinical trial budget template, which is provided to applicants in the Investigator Initiated Clinical Trials (IICT) program [12]. Finally, we chose a freely available international budgeting tool (identified in a scoping review conducted by our “MARTA” [Making Randomized Trials Affordable] group) [8] as a comparator (i.e., The Belgian Health Care Knowledge Centre (KCE) Budgeting Tool) [9, 10]. Other budgeting tools included in the scoping review that could be used to plan costs of an entire randomized clinical trial are either no longer freely available, focus on specific patient populations or interventions, or are less comprehensive than the KCE Budgeting Tool.

For each tool/approach, we collected data on the aim (calculation of full trial budget, estimation of services provided by CTU), the format of the tool (online tool, simple vs. programmed excel sheet, template/checklist), the procedure (consultation vs. independent use), availability of prespecified cost items (none, rough categories, detailed list), user-testing, the availability of an instruction manual, prespecified cost ranges for cost items, consideration of study design (sample size, number of arms, centers, and visits), consideration of fixed costs (costs independent of human resources), consideration of variable costs (human resource costs, e.g., time allocation for study tasks, unaccounted time), and indirect costs (overhead). Programmed excel sheets were defined as sheets with automatic calculation fields using underlying assumptions, whereas simple excel sheets only summed up cost items. Furthermore, we recorded if certain cost items were specifically listed in the budgeting tool. A total of 28 cost items organized into 12 domains were collected. A detailed list of all cost items can be found in Supplementary Material A.

Data were extracted for each tool by crosschecking the different domains and cost items. This was done by one reviewer (AG) and a second reviewer (MC, BS, or MB) carefully checked their consistency. In case of questions, institutions were contacted directly for clarification. The data was entered into an excel sheet.

### Analysis

We descriptively summarized the collected characteristics of the approaches/tools in tables and made qualitative comparisons across CTUs and the three funding agencies. Swiss CTUs were pseudonymized.

## Results

Of the nine included tools in our sample, six CTU tools and the KCE Budgeting Tool were based on excel sheets with varying level of detail (four consisted of simple cost item lists on a single sheet, three were more complex involving several sheets per trial). One CTU provided a list of bullet points and another CTU is working on turning their excel sheet into an online version. Three of the CTUs followed up their initial budget estimation with more precise cost estimates once the study details and CTU services were decided upon. In contrast, the SNSF provided an online tool to enter budget data (Table 1). One CTU tool and the Belgian KCE Budgeting Tool were publicly available. Three institutions were contacted to clarify questions about the form and structure of the tools and all institutions responded to all queries. The CTU tools can calculate budgets for commercial and non-commercial trials and used an activity-based budgeting approach.

**Table 1:** Overview of employed budget planning tools and approaches

Tools	Swiss budget tools								International budget tool
	CTU1	CTU2	CTU3	CTU4	CTU5	CTU6	CTU7	SNSF	Belgian Health KCE Tool (9,10**)**
Aim of tool	Estimate full trial budget	Budget for CTU services	Estimate full trial budget	Estimate full trial budget	Estimate full trial budget	Budget for CTU services	Estimate full trial budget	Estimate full trial budget	Estimate full trial budget
Procedure	Budget determined within the frame of a consultation	Budget determined within the frame of a consultation	Budget determined within the frame of a consultation	Budget determined within the frame of a consultation	Tool provided (possible assistance if needed)	Budget determined within the frame of a consultation	Budget determined within the frame of a consultation	Available to PIs applying for a grant	Available to PIs applying for a grant but can be used outside of program
Publicly available	No	No	No	No	Yes	No	No	No	Yes
Format	Checklist/bullet points	Simple excel sheet	Simple excel sheet	Empty excel sheet	Programmed excel sheet^a,b^	Programmed excel sheet^a,b^	Simple excel sheet	Checklist/online template	Programmed excel sheet^a^
Prespecified cost items	Detailed list	Detailed list	Detailed list	None	Detailed list	Detailed list	Detailed list	Rough categories	Detailed list
User-tested or based on experience	Based on experience	Based on experience	Based on experience	Based on experience	Based on experience	Based on experience	Based on experience	Unknown	Evidence for tool development with user-testing
Instruction manual	No	No	No	No	Yes	Yes	No	No	Yes
Examples of cost ranges	No	Hourly rates for services	No	No	Salary ranges	Rates of CTU provided	Salary ranges	Salary ranges	Yes, for some items, including hourly salary ranges
Consideration of study design									
Sample size	No	Yes	No	No	Yes	Yes	Yes	No	Yes
Arms	No	No	No	No	Yes	No	Yes	No	No
Centers	No	Yes	No	No	No	Yes	Yes	No	Yes
Visits	Yes	Yes	No	No	Yes	Yes	Yes	No	Yes
Direct fixed costs	Yes	No	Yes	No	Yes	No	Yes	Yes	Yes
Direct variable costs	Yes	Yes	Yes	No	Yes	Yes	Yes	Yes	Yes
Unaccounted time	Yes	Yes	No	No	Yes	No	Yes	No	No
Indirect costs	Yes	No	Yes	No	Yes	No	No	Yes	Yes

*Abbreviations:* *PI* Principal investigator, *CTU* Clinical Trial Unit, *KCE* Belgian Health Care Knowledge Centre

^a^Programmed sheet with some automatic calculation fields using underlying assumptions

^b^REDCap version announced

Including website, telephone hotline and videos

Five out of the seven CTUs provided an approach estimating the full trial budget. Two CTUs provided estimates for CTU services only (no overall trial costs). Most budget estimates provided by CTUs were only available in combination with a consultation session, guiding the trial investigators through the budgeting process (Table 1). Two CTUs provided tools, which could be used without consultation but offered further assistance if needed. None of the CTU tools was tested or provided plausible cost ranges for individual cost items; however, example salaries for study team members were provided in three CTU tools. Four tools considered both fixed and variable costs (personnel costs) in their budgeting approach.

The SNSF provided a simple online template, which is only accessible with an active grant application and is therefore not publicly available. The Belgian KCE Budgeting Tool has been developed with user feedback, is publicly available, provides a costing example, and supplies predefined cost ranges for some items. It is accompanied by an instruction manual, publication, and an option for support by contacting them through e-mail. The SNSF tool showed no evidence of testing or predefined cost ranges.

Six CTUs and the Belgian KCE Budgeting Tool provided a detailed list of cost items. The SNSF provided only rough categories. An overview of the cost is provided in Table 2.

**Table 2:** Costs items pre-specified in budget planning tools

	Swiss Budget tools								International Budget Tool
Tools	CTU1	CTU2	CTU3	CTU4	CTU5	CTU6	CTU7	SNSF	Belgian KCE tool [9, 10]
Infrastructure and material	Yes	Yes	Yes	No	Yes	No	Yes	Yes	Yes
Laboratory and Diagnostics									
Analysis	Yes	Yes	Yes	No	Yes	No	Yes	Yes	Yes
Shipment/sample transport	Yes	Yes	Yes	No	Yes	No	Yes	No	Yes
Sample storage	Yes	Yes	No	No	Yes	No	Yes	Yes	Yes
Imaging	Yes	Yes	Yes	No	Yes	No	No	Yes	Yes
Intervention									
Intervention cost	Yes	No	Yes	No	No	No	No	Yes	Yes
Control/placebo cost	Yes	No	Yes	No	No	No	No	Yes	Yes
IMPs/medical devices storage	Yes	No	Yes	No	Yes	No	Yes	No	Yes
Regulatory and documentation									
Submission process	Yes	Yes	Yes	No	Yes	Yes	Yes	Yes	Yes
Regulatory amendments	No	Yes	Yes	No	Yes	Yes	Yes	No	No
Site costs									
Set up of sites	Yes	No	Yes	No	Yes	No	Yes	No	Yes
Documentation at study site	Yes	Yes	Yes	No	Yes	No	Yes	No	Yes
Insurance	Yes	No	Yes	No	No	No	Yes	No	Yes
Recruitment and retention									
Screening	Yes	Yes	Yes	No	Yes	No	No	No	Yes
Dropouts	Yes	Yes	No	No	No	No	No	No	No
Patient compensation	Yes	No	Yes	No	Yes	No	Yes	No	Yes
Safety and quality assurance									
SAEs/AEs/SUSAR	Yes	Yes	Yes	No	Yes	No	Yes	No	No
Audits and inspections	Yes	No	Yes	No	No	Yes	No	No	Yes
Data Management									
Set-up, validation, maintenance of CRF	Yes	Yes	Yes	No	Yes	Yes	Yes	No	Yes
Data cleaning	No	Yes	Yes	No	No	Yes	No	No	Yes
Data sharing/data access committee	Yes	No	No	No	No	No	Yes	Yes	No
Monitoring									
Central monitoring	Yes	Yes	No	No	No	Yes	Yes	Yes	Yes
Site visits	No	Yes	Yes	No	No	Yes	Yes	No	Yes
Statistics									
Statistician	Yes	Yes	No	No	No	Yes	Yes	No	Yes
Sample size calculation	No	No	No	No	No	Yes	Yes	No	Yes
Interim and final analysis	No	No	Yes	No	No	Yes	No	No	Yes
Results dissemination									
Time for writing and dissemination of results	No	No	No	No	No	No	Yes	No	Yes
Publication fees/conferences fees	No	No	Yes	No	No	No	No	Yes	Yes

*Abbreviations:* *PI* principal investigator, *CTU* clinical trial unit, *KCE* Belgian Health Care Knowledge Centre, *IMPs* investigational medicinal products, *AE* adverse event, *SAE* serious adverse event, *SUSARs* suspected unexpected serious adverse reactions, *CRF* case report form

CTU 1 (21 of 28) and the Belgian KCE (24 of 28) budgeting tool considered the most cost items. The most reported cost items amongst CTU tools were costs for regulatory submission and costs for case report form set-up, validation, and maintenance (six from seven CTU tools). The least reported cost items were dissemination of results, publication, and conference costs (one of seven CTU tools) (Table 2). Cost items were estimated in a variety of different ways (as fixed costs, variable costs, or both); a detailed overview of how cost items were recorded can be found in the supplementary material (Table S1).

## Discussion

Our study showed that there is a large heterogeneity in budgeting approaches for clinical trials in Switzerland; none of the examined tools was user-tested, nor did they provide plausible cost ranges for individual cost items. This lack of validation and cost ranges was previously recognized on an international scale by a scoping review, investigating all publicly available budgeting tools [8]. The SNSF tool is only available to researchers with open grant applications. The Belgian KCE Budgeting Tool, in contrast, is more extensive and publicly available, was developed with user feedback, and provided an instruction manual [9, 10].

Analysis of the prespecified cost items revealed a focus of the CTUs on regulatory submission, quality management, data management, and monitoring, which aligns with services they provide to trialists. Costs related to the study conception and choice of the methods were not formally described but they can be more constantly estimated and fixed rates may be proposed more easily. In contrast, other cost driving categories such as participant recruitment were rarely mentioned, potentially furthering inaccuracies in trial budget planning [13]. This discrepancy may indicate divergent priorities of trial investigators and Swiss CTUs, which highlights the need for discussion between stakeholders about the purpose of budget tools that are used at a CTU level.

Future projects should test the approaches used by CTUs and discuss working towards a standardized set of core items. This effort could help improve national budgeting practices by providing insights into actual vs. budgeted costs, contributing to improved clinical research, and reduced research waste. The SNSF could take a leading role in providing a comprehensive costing tool. This tool should include user-testing and predefined cost ranges for various items, facilitating adequate funding. To achieve the latter, an ongoing project is systematically collecting empirical costs and resource use data for the planning, conduct, and finalization of randomized clinical trials in Switzerland, Germany, and the UK [14]. These empirical data may help trial investigators, CTUs, and funders to improve the accuracy of trial budget estimations.

In the broader context of clinical trial costs, it is important to note that CTU budgeting is not the primary cause of underfunded trials. Research budgets are inherently constrained by funding limits, requiring researchers to adapt their trial plans accordingly. Additionally, lengthy approval processes, extended setup times, and recruitment challenges significantly impact trial timelines and budgets, underscoring the need for comprehensive solutions beyond costing tools.

This study has several limitations. Firstly, the tools/approaches were not used or tested on actual clinical trial budgets, i.e., their accuracy in providing a reliable budget estimation is yet to be determined. Secondly, the tools/approaches provided by CTUs were mainly for intra-institutional use and might, therefore, be tailored to the individual needs of the CTUs, which could explain institutional variations.

## Conclusion

In conclusion, there is substantial heterogeneity in the current approaches to estimate clinical trial budgets in Switzerland. None of the tools/approaches was empirically tested or provided cost ranges. Stakeholders should develop a standardized set of cost items and validate budgeting approaches across Switzerland.

## Supplementary Information

Supplementary Material 1.

## Data Availability

Data can be shared upon request.
