# Supplementary material for: Clinical trial budgeting approaches in Switzerland—a meta-research study
Source: Trials. 2025 May 14;26:158. doi: 10.1186/s13063-025-08855-1 (PMC12079863; doi:10.1186/s13063-025-08855-1)
Supplement: Supplementary file 1 — Supplementary Material 1. [file 13063_2025_8855_MOESM1_ESM.docx]

**Supplementary Material: Clinical Trial Budgeting Tools in Switzerland - a meta-research study**

# **Authors**

Alexandra Griessbach ^1^ *, Malena Chiaborelli ^1,2^, Klaus Ehrlich ^3^, Regina Grossmann ^3^, María De Medina Redondo ^3^, Aurélie Fayet ^4^, Reinhard Maier ^5^, Sven Trelle ^6^, Angèle Gayet-Ageron ^7^, Alessandro Ceschi ^8^, Benjamin Speich ^1 †^, Matthias Briel ^1 †^

**A) Detailed List of collected Items:**

This included costs for infrastructure, (material and shipment), laboratory and diagnostics (analysis, transport and storage), imaging, intervention costs (intervention, placebo/control and storage of intervention), regulatory costs (submission and amendments), site costs (site set up, documentation and insurance), recruitment and retention costs (patient compensation, screening costs, drop outs), costs for safety and quality assurance (costs for adverse events [AE], serious adverse events [SAE] and suspected unexpected serious adverse reaction [SUSARS] in addition to audits), data management (database setup, cleaning and data access committees), monitoring (site visits and central monitoring), statistics (sample size calculation, interim and final analyses), communication and results dissemination costs.

| **Table S1:** **Metric used to estimate the budget by items** | | | | | | | | |
| --- | --- | --- | --- | --- | --- | --- | --- | --- |
|  | **Swiss Budget tools** | | | | | | | |
| **Tools** | **CTU1** | **CTU2** | **CTU3** | **CTU4** | **CTU5** | **CTU6** | **CTU7** | **SNSF** |
| **Infrastructure and material** | as bullet point | Minutes for task | Fixed cost for task | No | fixed cost per unit | No | as fixed costs per year | as fixed costs per year |
| **Laboratory and Diagnostics** | as bullet point | Minutes for task | Fixed cost for task | No | fixed cost per unit | No | No | No |
| laboratory analysis | as bullet point and as fixed cost per visit | Minutes for task | Fixed cost for task | No | fixed cost per unit | No | as an hourly rate | as fixed costs per year |
| Shipment/ sample transport | as bullet point | Minutes for task | Fixed cost for task | No | in hours per team role | No | as an hourly rate | No |
| sample storage | as bullet point | Minutes for task |  | No | in hours per team role and fixed cost | No | as an hourly rate | as fixed costs per year |
| **Imaging** | as bullet point and as fixed cost per visit | Minutes for task | Fixed cost for task | No | fixed cost per unit | No | No | as fixed costs per year |
| **Intervention** |  |  |  |  |  |  |  |  |
| costs of experimental intervention | as bullet point | No | Fixed cost for task | No | No | No | No | as fixed costs per year |
| costs of control/placebo intervention | as bullet point | No | Fixed cost for task | No | No | No | No | as fixed costs per year |
| storage of IMPs/medical devices | as bullet point | No | Fixed cost for task | No | fixed cost per unit | No | as an hourly rate | No |
| **Regulatory and documentation** |  |  |  |  |  |  |  |  |
| submission process | as bullet point | Minutes for task | Fixed cost for task | No | in hours per team role and fixed cost | in time and fixed costs per hour | as fixed costs per year | as fixed costs per year |
| regulatory amendments | No | Minutes for task | Fixed cost for task | No | in hours per team role | in time and fixed costs per hour | as fixed costs per year |  |
| **Site costs** |  |  |  |  |  |  |  |  |
| set up of sites | fixed startup fee | No | Fixed cost for task | No | in hours per team role and fixed cost | No | as an hourly rate | No |
| documentation at study site | mention IC, contracts, protocol | Minutes for task | Fixed cost for task | No | in hours per team role | No | as an hourly rate | No |
| insurance | as bullet point | No | Fixed cost for task | No | No | No | as fixed costs per year | No |
| **Recruitment and retention** |  |  |  |  |  |  |  |  |
| screening | as bullet point | Minutes for task | Fixed cost for task | No | in hours per team role | No | No | No |
| drop outs | as bullet point | Minutes for task | No | No | No | No | No | No |
| patient compensation | as bullet point | No | Fixed cost for task | No | fixed cost per unit | No | as fixed costs per year | No |
| **Safety and quality assurance** |  |  |  |  |  |  |  |  |
| SAEs/AEs/SUSARs | as bullet point | Minutes for task | Fixed cost for task | No | in hours per team role | No | as an hourly rate | No |
| audits and inspections | as bullet point | No | Fixed cost for task | No | No | in time and fixed costs per hour | No | No |
| **Data Management** |  |  |  |  |  |  |  |  |
| database set-up validation, maintenance | as bullet point manual | Minutes for task | Fixed cost for task | No | in hours per team role | in time and fixed costs per hour | as an hourly rate | No |
| data cleaning | No | Minutes for task | Fixed cost for task | No | No | in time and fixed costs per hour | No | No |
| Data Access Committee | as bullet point | No | No | No | No | No | as fixed costs per year | as fixed costs per year |
| **Monitoring** |  |  |  |  |  |  |  |  |
| central monitoring | as bullet point | Minutes for task | No | No | No | in time and fixed costs per hour | as fixed costs per year | as fixed costs per year |
| site visits | No | Minutes for task | Fixed cost for task | No | No | in time and fixed costs per hour | as an hourly rate | No |
| **Statistics** |  |  |  |  |  |  |  |  |
| sample size calculation | No | No | No | No | No | in time and fixed costs per hour | as an hourly rate | No |
| interim analysis and final analysis | No | Minutes for task | Fixed cost for task | No | No | in time and fixed costs per hour | No | No |
| **Communication and dissemination** |  |  |  |  |  |  |  |  |
| dissemination of results | No | No | No | No | No | No | as fixed costs for each year | No |
| publication / conferences | No | No | Fixed cost for task | No | No | No | No | as fixed costs per year |
| *Abbreviations:* IMPs: Investigational Medicinal Products, AE: Adverse Event, SAE: Serious Adverse Event, SUSARs: Suspected Unexpected Serious Adverse Reactions, CRF: Case Report Form | | | | | | | | |
